# Extracellular vesicles of *Trypanosoma cruzi* tissue-culture cell-derived trypomastigotes: Induction of physiological changes in non-parasitized culture cells

**DOI:** 10.1371/journal.pntd.0007163

**Published:** 2019-02-21

**Authors:** Lissette Retana Moreira, Fernando Rodríguez Serrano, Antonio Osuna

**Affiliations:** 1 Instituto de Biotecnología, Grupo de Bioquímica y Parasitología Molecular, Departamento de Parasitología, Universidad de Granada, Granada, Spain; 2 Instituto de Biopatología y Medicina Regenerativa, Universidad de Granada, Granada, Spain; University of Texas at El Paso, UNITED STATES

## Abstract

**Background:**

*Trypanosoma cruzi* is the obligate intracellular parasite that causes Chagas disease. The pathogenesis of this disease is a multifactorial complex process that involves a large number of molecules and particles, including the extracellular vesicles. The presence of EVs of *T*. *cruzi* was first described in 1979 and, since then, research regarding these particles has been increasing. Some of the functions described for these EVs include the increase in heart parasitism and the immunomodulation and evasion of the host immune response. Also, EVs may be involved in parasite adhesion to host cells and host cell invasion.

**Methodology/Principal findings:**

EVs (exosomes) of the Pan4 strain of *T*. *cruzi* were isolated by differential centrifugation, and measured and quantified by TEM, NTA and DLS. The effect of EVs in increasing the parasitization of Vero cells was evaluated and the ED50 was calculated. Changes in cell permeability induced by EVs were evaluated in Vero and HL-1 cardiomyocyte cells using cell viability techniques such as trypan blue and MTT assays, and by confocal microscopy. The intracellular mobilization of Ca^2+^ and the disruption of the actin cytoskeleton induced by EVs over Vero cells were followed-up in time using confocal microscopy. To evaluate the effect of EVs over the cell cycle, cell cycle analyses using flow cytometry and Western blotting of the phosphorylated and non-phosphorylated protein of Retinoblastoma were performed.

**Conclusion/Significance:**

The incubation of cells with EVs of trypomastigotes of the Pan4 strain of *T*. *cruzi* induce a number of changes in the host cells that include a change in cell permeability and higher intracellular levels of Ca^2+^ that can alter the dynamics of the actin cytoskeleton and arrest the cell cycle at G0/G1 prior to the DNA synthesis necessary to complete mitosis. These changes aid the invasion of host cells and augment the percentage of cell parasitization.

## Author summary

Extracellular vesicles (EVs) are a diverse group of nanoparticles involved in intercellular communication under physiological and pathological conditions. *Trypanosoma cruzi*, the protozoan that causes Chagas disease, releases EVs that facilitate parasite invasion of the host cell, immunomodulate the host response, and help the parasite to evade this response. However, little is known about how the host cell is altered. In this work, we confirm that EVs of tissue-culture cell-derived trypomastigotes of the Pan4 strain increase cell parasitism. We also demonstrate that EVs affect cell permeability in Vero cells and cardiomyocytes and raise intracellular Ca^2+^ levels, altering the actin filaments and arresting the cell cycle at the G0/G1 phases. This work seeks to elucidate the way in which EVs influence certain aspects of the cell physiology that favour the establishment of this parasite inside the host cell.

## Introduction

*Trypanosoma cruzi* is an intracellular protozoan parasite that causes Chagas disease or American trypanosomiasis. An estimated 8 million people are infected with this parasite worldwide, with some 300,000 new cases and 15,000 deaths annually [1]. *T*. *cruzi* has a life cycle that includes mammals and blood-sucking bugs (Hemiptera, Reduviidae) as hosts. Humans can be infected through the insects faeces, by vertical (congenital) transmission, transmission by blood transfusions, organ transplants, or oral contamination via tainted fluids and foods [2].

Chagas disease displays symptomatic and pathological variations among infected individuals [3] but is characterized by an acute as well as a chronic stage. During the chronic stage, approximately 30% of the patients develop significant complications, which may include megasyndromes of the gastrointestinal tract (such as megacolon or megaesophagus), neurological complications, and cardiomyopathy [4–7].

The pathogenesis of Chagas disease is a multifactorial process. The molecular invasion mechanisms by *T*. *cruzi* trypomastigotes (T) and the associated regulatory pathways have been intensely investigated for many years [8]. A large number of molecules have been involved and are described as part of the secretome of *T*. *cruzi* [9]. Some of them are included in extracellular vesicles (EVs). EVs are small membrane-bound vesicles classified based on their size, biogenesis, and composition [10] in: a) exosome-like EVs (20–100 nm), b) ectosome-like EVs (100–1000 nm) and c) apoptotic blebs (>1000 nm) [9,11].

The presence of EVs of *T*. *cruzi* was first described in 1979 by da Silveira et al., who demonstrated the secretion of plasma-membrane vesicles by *T*. *cruzi* epimastigote forms [12]. These vesicles were later detected by Gonçalves et al. (1991) in host-cell-derived trypomastigotes [13]. Since then, numerous publications concerning EVs have appeared, demonstrating their role in cell-to-cell communication, pathogenesis, evasion of the immune response and diagnosis.

The cargo of EVs of *T*. *cruzi* contain proteins involved in host-parasite interactions, signalling, trafficking, and membrane fusion, transporters, oxidation-reduction, etc. [9]. Small RNAs derived from tRNAs and rRNAs have also been reported [14]. Some of the functions described for these EVs include the increase in heart parasitism and the immunomodulation of the host response [15]; the evasion of innate immunity [16]; and the induction of the release of EVs by the host blood cells that are involved in inhibiting complement-mediated lysis [17]. Also, EVs may be involved in parasite adhesion to host cells and host-cell invasion [15,17–20]. Recently, EVs have proved useful in evaluating disease severity as well as vaccine and drug effectiveness against chagasic cardiomyopathy [21]. However, little is known about the capacity of EVs to modulate the host-cell conditions. In this sense, the present work seeks to elucidate certain effects exerted by *T*. *cruzi* EVs over the parasite establishment inside the host cell.

## Methods

### Cell cultures, parasite strains, and isolation of EVs

Vero (ECACC 84113001) and 3T3 cells (CRL 1658) were cultured in Nunc cell-culture flasks of 75 cm^2^ surface area (Thermo Fischer Scientific, USA) in Modified Eagle’s Medium (MEM) (Sigma, USA) supplemented with 10% foetal bovine serum (Gibco, USA) previously inactivated at 56°C for 30 min (IFBS) plus antibiotics (penicillin 100 U/mL, streptomycin 100 μg/mL). The cultures were maintained at 37°C, in a moist atmosphere enriched with 5% CO_2_. HL-1 cardiac muscle-cell line was grown as described above, using Claycomb medium supplemented with 10% IFBS, norepinephrine 0.1 mM, L-glutamine 2 mM and antibiotics (penicillin 100 U/mL, streptomycin 100 μg/mL). The cell cultures were routinely monitored for *Mycoplasma* by PCR.

Vero cells were initially infected with purified metacyclic trypomastigotes of the Pan4 (Tc Ia + Tc Id) strain of *T*. *cruzi* obtained *in vitro*, according to de Pablos et al. (2011) [22]. After 120 h of the intracellular development of the parasite, tissue-culture cell-derived trypomastigotes (TcT) were harvested by centrifugation. Parasites were collected routinely every 120 h from the infected cell monolayer. The culture medium was centrifuged at 3,000 xg for 5 min and the pellet with the parasites was washed in PBS four times.

To obtain the EVs from the TcT, we followed the procedure described previously by de Pablos et al. (2016) [18], with some modifications. Parasites were incubated for 5 h at 37°C in RPMI medium (Sigma, USA) buffered with 25 mM HEPES at 7.2 and supplemented with 10% exosome-free IFBS. Afterwards, parasites were removed by centrifugation at 3,500 xg for 15 min and the supernatant was collected and centrifuged at 17,000 xg for 30 min at 4°C. This supernatant was filtered through a 0.22 μm pore filter (Sartorius, Germany) and ultracentrifuged at 100,000 xg for 16–18 h to obtain the EVs (mostly exosomes). All the steps were performed in an ultracentrifuge Avanti J-301 (Beckman Coulter, USA) with a JA-30.50 Ti rotor. The resulting pellet was washed three times in PBS in an ultracentrifuge Sorwal WX80 (Thermo Fisher Scientific, USA) with F50L-24 x 1.5 fixed-angle rotor and resuspended in 100 μL PBS.

The isolation procedure was evaluated by Transmission Electron Microscopy (TEM), Nanoparticle Tracking Analysis (NTA) and Dynamic Light Scattering (DLS). The proteins from the EVs were quantified using the Micro-BCA protein assay kit (Thermo Fischer Scientific, USA), using bovine-serum albumin as standard. Viability of the TcT after shedding of EVs was evaluated using the trypan blue exclusion test. After 5 h, no significant death was detected and over 99% of the parasites were viable.

To demonstrate the specificity of the effects of the EVs from the TcT of *T*. *cruzi* and to evaluate the effect of the EVs of trypomastigotes of the Pan4 strain over the infection of cells with *T*. *cruzi* from a different DTU and another intracellular microorganism, we performed the isolation of EVs from *Crithidia mellificae* and the 3T3 cell line (a fibroblast cell line) and evaluated the effect of these EVs over the parasitization percentages of cells infected with *T*. *cruzi* Pan4. We also employed *T*. *cruzi* 4162 strain (Tc IV) and tachyzoites of *Toxoplasma gondii* RH for the infection of cells previously incubated with EVs of *T*. *cruzi* Pan4.

For the isolation of EVs from choanomastigotes of *Crithidia mellificae*, 1x10^7^ parasites were incubated for 24 h at 28°C in LIT medium. Nunc cell-culture flasks of 75 cm^2^ surface area (Thermo Fischer Scientific, USA) with confluent monolayers of 3T3 cells were washed 3 times with MEM without IFBS and the cells were incubated for 24 h at 37°C with MEM (Sigma, USA). After 24 h of incubation, the culture media were collected and centrifuged at 3,500 xg for 15 min and the obtained supernatants were handled the same way as for the isolation of EVs from TcT. DLS and the quantification of the protein concentration of these samples using the Micro-BCA protein assay kit (Thermo Fischer Scientific, USA) were performed as described above.

The purification of metacyclic trypomastigotes of *T*. *cruzi* 4162 strain was also performed according to de Pablos et al. (2011) [22] and TcT were obtained after the infection of Vero cells as described. Tachyzoites of *Toxoplasma gondii* RH strain were maintained in our laboratory by serial passage, in semiconfluent monolayers of Vero cells and cultured in the same conditions as *T*. *cruzi*. The egressed parasites were harvested, centrifuged at 5,000 xg for 10 min, washed three times in PBS and added to the cell cultures in a ratio 5:1 (parasites:cell).

### Transmission electron microscopy

To confirm the presence of EVs in our samples, we resuspended an aliquot of the pellet in 0.1 M Tris HCl (pH 7.2) and 5 μL each sample were adsorbed directly onto Formvar/carbon-coated grids and stained with 2% (vol/vol) uranyl acetate, for the direct observation in a TEM, LIBRA 120 PLUS Carl Zeiss microscope. The diameter of the EVs was measured by ImageJ 1.41 software.

### Nanoparticle Tracking Analysis (NTA) and Dynamic Light Scattering (DLS)

Distribution, size, and concentration of *T*. *cruzi* EVs from trypomastigotes was determined by measuring the rate of Brownian motion according to the particle size, using a Nanosight NS300 (Malvern Instruments, UK). This system was equipped with a sCMOS camera and a blue 488 nm laser beam. Samples were diluted 1/100 just before the analysis, in low-binding Eppendorf tubes with PBS and the measurements were performed at 25°C. For data acquisition and information processing, we used the NTA software 3.2 Dev Build 3.2.16. The particle movement was analysed by NTA software with the camera level at 16, slider shutter at 1200, and slider gain at 146.

To confirm the results obtained by NTA, we also performed Dynamic Light Scattering (DLS) of the EVs of trypomastigotes, choanomastigotes and cells using a Zetasizer nano ZN90 (Malvern Instruments, UK). Samples were prepared the same way as described for the NTA and the measurements were also performed at 25°C. For data acquisition and information processing, the Zetasizer Ver. 7.11 software was employed.

### Western blotting of EVs of *T*. *cruzi* Pan4 to evaluate the presence of cruzipain, trans-sialidase (TS) and MASPs (SP)

The presence of some molecules without orthologues in other organism and involved in the invasion process of *T*. *cruzi* was evaluated in EVs by Western blotting. Briefly, 300 μg of EVs isolated from TcT of the Pan4 strain were resolved by SDS-PAGE, transferred to a nitrocellulose membrane and blocked overnight with 5% non-fat milk in PBS-0.1% Tween 20. Primary antibodies anti-cruzipain (1:3,000), anti-TS (mAb 39) (1:1,000), and anti-MASPs (signal peptide, SP) (1:1,000) [16] were incubated overnight at 4°C. The membranes were washed with PBS-0.1% Tween 20 and incubated for 1 h with goat anti-mouse IgGs conjugated with peroxidase (1:1,000) (Dako Agilent Pathology Solutions, USA) in the case of TS and MASPs and goat anti-rabbit IgGs conjugated with peroxidase (1:2,000) in the case of cruzipain (Dako Agilent Pathology Solutions, USA). The reaction was visualized using Clarity ECL Western substrate (BioRad, Spain) in a ChemiDoc Imaging system (BioRad, Spain).

### Optimization of EV-cell incubation conditions and invasion assays

Cultures of 5x10^4^ Vero cells were grown in MEM supplemented with 10% IFBS over round 13-mm coverslips (Marienfeld, Germany), in Nunc 24-well plates (Thermo Fischer Scientific, USA) for 24 h at 37°C and 5% CO_2_. After this time, coverslips with cells were washed 3 times in MEM and incubated for 2 h with 0.1, 0.25, 0.5, 1 and 2.5 μg/mL EVs in MEM. After the incubation, cells were infected with *T*. *cruzi* trypomastigotes of the Pan4 strain, at a parasite:host cell ratio of 5:1. After 4 h, parasites were removed and the cells were washed and maintained in culture for 24 h. The cultures were fixed with methanol and stained with Giemsa. Parasitization percentages and parasitization indexes (number of amastigotes per cell) were calculated after counting at least 400 cells.

The invasion assays were also performed using *T*. *cruzi* EVs submitted to thermal and chemical treatments. For the thermal treatments, EVs were incubated in a water bath at 50°C, 70°C, and 90°C for 30 min. For the chemical treatments, EVs were incubated with the proteolytic enzymes trypsin (0.5 mg/mL) and proteinase K (0.5 mg/mL) for 1 h at 37°C and with sodium periodate (10 mg/mL) for 20 h at room temperature, in the dark, to reduce the glycoconjugates surrounding the EVs. After the treatments, EVs were washed twice in PBS by ultracentrifugation at 100,000 xg for 1 h, incubated with the Vero cell cultures for 2 h. The protocol for cell infection was followed as described above.

The specificity of the effects of the EVs isolated from trypomastigotes of *T*. *cruzi* Pan4 and the effect of EVs from TcT of the Pan4 strain over the invasion of another *T*. *cruzi* strain and intracellular parasite were evaluated. For these experiments, cultures of 5x10^4^ Vero cells were grown the same way as described for the invasion assays using the Pan4 strain. The cells were incubated for 2 h at 37°C with 0.38 μg/mL EVs from *Crithidia mellificae* or 3T3 cells. After this time, the cells were infected with *T*. *cruzi* trypomastigotes of *T*. *cruzi* Pan4 (parasite:host cell ratio of 5:1) and after 4 h of interaction, the parasites were removed. The cells were washed and maintained in culture for 24 h, when they were fixed with methanol and stained with Giemsa. Parasitization percentages and indexes were calculated.

The effect of EVs of the Pan4 strain of *T*. *cruzi* over the infection of cells with trypomastigotes of *T*. *cruzi* 4162 strain (Tc IV) and tachyzoites of *T*. *gondii* RH were performed. In this case, cells were incubated for 2 h with EVs of *T*. *cruzi* Pan4 and then infected with trypomastigotes of *T*. *cruzi* 4162 strain or tachyzoites of *T*. *gondii* RH in a parasite:cell ratio of 5:1. After 4 h, the parasites were removed, the cells were washed and maintained in culture for 24 h. Parasitization percentages and indexes were calculated after the evaluation of the cells by Giemsa stain.

### Changes in cell permeability by *T*. *cruzi* EVs

To assess the potential capacity of EVs to permeabilize cells, we cultured 5 x 10^4^ Vero cells in 12-well plates as described above. The potential changes in permeability during or after the EVs-cell interaction was evaluated using the *Aspergillus giganteus* ribotoxin α-sarcin, a ~17 kDa protein that inhibits protein biosynthesis when the cells are previously permeabilized [23–25]. Briefly, after 24 h of culture, cells were washed 3 times with MEM and incubated with 0.38 μg/mL EVs in MEM for 2 h. Cells were washed once and 20 μM of α-sarcin (Sigma, USA) was added for 4 h. After this time, cells were washed 4 times and subsequently incubated with MEM supplemented with 10% IFCS. In a parallel assay, the EVs and α-sarcin were added simultaneously to the cell culture. Viability of the cells was determined using the trypan blue exclusion test as well as the MTT viability assay (Sigma, USA). Cell viability was also determined after the incubation of the cell cultures with EVs, α-sarcin and cells without any treatment as negative controls.

The HL-1 cell line was derived from atrial cardiomyocytes is a cell line that maintains a series of cardiac characteristics such as morphological, biochemical, and electrophysiological properties *in vitro* [26]. On round 13-mm coverslips, 5x10^4^ cells were grown in Claycomb medium supplemented with 10% IFBS, norepinephrine 0.1 mM, L-glutamine 2 mM and antibiotics. After 24 h of culture, cells were washed and incubated with 0.38 μg/mL EVs in MEM for 2 h. Afterwards, coverslips were washed 3 times and fixed with a solution of 2% paraformaldehyde and 1% glutaraldehyde for 1 h, washed 3 times in PBS and blocked with a solution containing 1% BSA and 0.3 M glycine in PBS, for 1 h. Cells were washed 3 times and incubated with an anti-β2-adrenergic receptor primary antibody (1:500) (Thermo Fisher Scientific, USA) for 1 h. Afterwards, cells were washed 3 times and incubated in the dark, with a goat anti-rabbit IgG antibody conjugated with Alexa Fluor 647 (1:500) (Thermo Fisher Scientific, USA) for 1 h, at 37°C. Finally, samples were washed 4 times, mounted in Vectashield mounting medium with DAPI (Vector Laboratories, USA) and imaged with a Leica DM5500B inverted microscope (Leica Microsystems, Germany).

HL-1 cells cultured and fixed as described above but treated with a solution of NP-40 in 10 mM citric acid (pH 6) were employed as positive control of permeabilization of the assay.

### Intracellular mobilization of Ca^2+^ induced by *T*. *cruzi* EVs

Vero cells were grown overnight in MEM without phenol red plus IFBS and in MEM without phenol red plus IFBS and 2.5 μM EDTA, on μ-slide ibidi multichamber dishes. Cells were washed 3 times in MEM without phenol red and incubated at 37°C for 20 min with Fluo4-AM (Thermo-Fisher, USA) in 1) MEM without phenol red, 2) MEM without phenol red plus 2.5 μM EDTA and 3) a culture medium similar to MEM but without Ca^2+^ and Mg^2+^. Fluo-4 is an indicator that exhibits greater fluorescence upon binding intracellular free Ca^2+^. It presents an AM grouping (acetoxymethyl ester) that, when internalized, is cleaved by intracellular esterases and released to bind to cytoplasmic calcium [20].

After 20 min of incubation of the cells with Fluo-4 AM, EVs of TcT of *T*. *cruzi* Pan4 were added to the cells and followed-up in time until 25 min of interaction. Basal controls of fluorescence in cells before the application of the stimulus with EVs were included.

Images were taken every 5 min with a confocal microscope Nikon A1 (Nikon Instruments, The Netherlands) equipped with 10x, 20x multi-immersion, 40x oil, 60x oil, and 60x water objectives and a system of cell incubation at 37°C with enriched atmosphere with 5% CO_2_. The Fluo4 probe was excited at 494–506 nm and light emission was detected at 516 nm. The fluorescence intensity was analysed and normalized with reference to the basal fluorescence using NIS Elements Software (Nikon Instruments, The Netherlands). The analysis of images was performed using Fiji software (Fiji is Just Image J). Controls of cells incubated with A23187 (a calcium ionophore) and 3-isobutyl-1-methylxantine (IBMX) (a non-specific inhibitor of cAMP and cGMP phosphodiesterase that induces calcium release from intracellular stores) were included.

### Effects of the EVs of *T*. *cruzi* over the actin cytoskeleton integrity

Cultures of 5x10^4^ Vero cells were seeded over round 13-mm coverslips (as described above) and allowed to attach to the coverslips overnight. The cells were washed 3 times with MEM and incubated in MEM during different times with 0.38 μg/mL EVs in MEM. After this incubation step, coverslips were washed 3 times and fixed with cold acetone (Scharlab, Spain) for 15 min at -20°C. After the fixation step, coverslips were washed 3 times with PBS and permeabilized in a solution of 0.1% Triton X-100 (Sigma, USA) for 10 min. The cells were washed 3 times and blocked with 1% BSA and 0.3 M glycine in PBS for 1 h in PBS. After this time, the cells were washed and incubated with 5 μg/mL of a vimentin polyclonal antibody (1:300) (Thermo Fischer Scientific, USA) for 1 h, washed 4 times and incubated in the dark, with a goat anti-rabbit IgG antibody conjugated with Alexa Fluor 647 (1:500) (Thermo Fisher, USA) for 1 h, at 37°C. The coverslips were washed 4 times and stained with a solution of phalloidin, fluorescein isothiocyanate labelled (Sigma, USA) for 30 min. Samples were finally mounted in Vectashield mounting medium with DAPI (Vector Laboratories, USA) and imaged in a Leica DM5500B inverted microscope (Leica Microsystems, Germany). Images were captured 15 min, 30 min, 120 min, and 24 h after EVs-cell contact. Cells not treated with EVs and cells incubated with the final supernatant from the EVs purification medium were employed as controls.

### Cell cycle analysis: Flow cytometry and Western blotting of the phosphorylated and non-phosphorylated protein of Retinoblastoma (pRb)

Vero cells (1x10^5^) were synchronized according to the method described previously by Osuna et al. (1984) [27]. Cells were seeded in 6-well plates with a culture medium with 25 mM thymidine for 12 h, when the medium was replaced with MEM + 10% IFCS. Afterwards, cells were washed with MEM and 1 h later, EVs were added directly to each well. One hour after this EV-cell contact, cells were washed and maintained for 2 and 8 h. Afterwards, the culture medium of the corresponding wells was removed, the cells were washed with PBS, fixed with 70% cold ethanol and incubated with a solution (0.2 M Na_2_HPO_4_, 0.1 M citric acid, pH 7.8) for 15 min at 37°C. Cells were then centrifuged, washed with PBS and resuspended in 250 mL of a solution of propidium iodide (40 mg/mL) and RNAse (100 mg/mL) for 30 min at 37°C in the dark, according to Carrasco et al. (2014) [28]. Finally, the samples were analysed in a FACS Calibur (BD Biosciences, San Jose, CA, USA) flow cytometer. The results were analysed with FlowJo software (v 7.6.5, Tree Star, Inc.).

Phosphorylation of the protein Rb after the incubation of cells with EVs was evaluated by immunoblotting. Briefly, 1x10^5^ cells were grown in 6-well plates for 24 h. Cells were washed with MEM and incubated with EVs for 5, 10, 30 and 60 min. After this session, the cells were washed with PBS and lysed in RIPA lysis buffer with a protease inhibitor cocktail (Roche, Switzerland) for 15 min. Cells were harvested with a cell scraper and centrifuged at 14,000 xg for 10 min at 4°C. Supernatants were transferred to new Eppendorf tubes and stored at -20°C. The protein from cell lysates was quantified using the Bradford reagent (Sigma, USA) and 90 μg of protein from cell lysates were resolved by SDS-PAGE, transferred to a nitrocellulose membrane and blocked overnight with 5% non-fat milk in PBS-0.1% Tween 20. Rb (1:2,000) and phospho-Rb (1:1,000) (Cytoskeleton, USA) primary antibodies (Sigma, USA) were incubated overnight at 4°C. Tubulin antibody (1:5,000) (Cytoskeleton, USA) was used as the loading control. The membranes were washed with PBS-0.1% Tween 20 and incubated for 1 h with goat anti-mouse IgGs conjugated with peroxidase (1:1,000) (Dako Agilent Pathology Solutions, USA) in the case of Rb, goat anti-rabbit IgGs conjugated with peroxidase (1:2,000) in the case of phospho-Rb (Dako Agilent Pathology Solutions, USA), and rabbit anti-sheep IgGs conjugated with peroxidase (1:5,000) (Dako Agilent Pathology Solutions, USA) in the case of tubulin. The reaction was also visualized using Clarity ECL Western substrate (BioRad, Spain) in a ChemiDoc Imaging system (BioRad, Spain).

### Statistical analysis

Quantification values represent the means of two or more independent experiments, each performed in triplicate. Means and standard deviations of the EVs size (NTA), percentage of infected cells (invasion assays) and percentage of live cells (permeabilization assays) were calculated. One-factor ANOVAs were performed to detect significant differences between cells treated with EVs and control cells in the case of permeabilization and cell-cycle assays. Multiple *post hoc* comparisons were performed using the Tukey-Kramer test on GraphPad Prism 5 Software (USA). Values with p<0.0001 were considered statistically significant (***).

## Results

### Transmission electron microscopy, NTA and DLS of EVs of *T*. *cruzi*

After the incubation of 1x10^7^ trypomastigotes of the Pan4 strain for 5 h at 37°C in the culture medium for the release of EVs, 12 μg of total protein of EVs were obtained. The isolation of the EVs by the ultracentrifugation protocol described was evaluated by TEM, NTA and DLS and the presence of surface molecules of *T*. *cruzi* in these samples was confirmed by Western blotting (S1 Fig).

Most of the particles visualized by negative staining under TEM were of 30–100 nm size (S1A Fig). Analyses by NTA revealed a majority of EVs with a size of 70.7 ± 7.3 nm (S1C Fig) and a concentration of approximately 5x10^10^ ± 3.9x10^9^ particles/mL. In the DLS analyses, two populations of EVs with different sizes were observed for TcTs, choanomastigotes and 3T3 cells (S1D, S1E and S1F Fig); EVs of *T*. *cruzi* showed a population of 23.05 ± 6.96 nm and a population of 55.74 ± 13.97 nm (S1D Fig). Western blotting confirmed the presence of cruzipain, trans-sialidase and MASPs (SP) in the EVs of *T*. *cruzi* Pan4 (S1B Fig).

### Invasion assays

To evaluate the effect of EVs of TcT from the Pan4 strain in host-cell invasion, we tested different doses measured in total μg/mL of protein. After 2 h of incubation of the cells with the EVs, the infection with T was performed in a ratio 5:1 (T:cell) for 4 h. Counts were performed 24 h later, as described in the Methods section. The results indicate that the parasitism increased the most when the cells were treated with 0.5 μg/mL of EVs. These values in percentage of parasitization (88.88 ± 3.73) significantly differed with respect to the other doses analysed (S2A Fig). From these results, the Effective Dose 50 (ED50) for subsequent trials was set as 0.38 μg/mL.

Regarding the effect of EVs on cells over time (the increase in cell parasitization), these were treated with 0.38 μg/mL of total protein of EVs for 2 h. After the cells were washed three times in medium without serum, they were infected at different time points after the treatment with EVs at a T:cell ratio 5:1, as described above. The results (S2B Fig) show that the difference between the percentage of parasitization of the cells treated with the EVs vs. the percentage of parasitization of non-treated control cells were statistically significant up to 8 h after the treatment. The effects were more evident at 2 and 4 h, when the increase in the percentages was higher compared to untreated cultures. This effect was not appreciated when the cells were infected 24 h after the treatment with the EVs. The parasitization indexes (number of parasites per cell) calculated also differed. For example, in the case of the cells incubated with EVs for 2 h was 2.78 ± 0.55, an index that was twice the parasitization index of the control infected cells without the previous treatment (1.33 ± 0.18).

A series of experiments were performed to study whether the thermal treatment, the treatment with proteolytic enzymes or the reduction of the glycoconjugates surrounding the EVs alters their ability to induce higher levels of parasitization in the host cells with which they interact. Results are shown in S2C and S2D Fig. The thermal treatment of the EVs at 50°C, 70°C and 90°C annuled the action of the EVs on the cells, so the increase in the percentage of parasitization was not observed. The enzymatic treatment with the two proteases employed and the treatment with sodium periodate (for the reduction in the content of carbohydrates of the EVs) also inhibited the capacity of increasing the cell parasitization by trypomastigotes of *T*. *cruzi* in the cultures.

**Fig 1:**
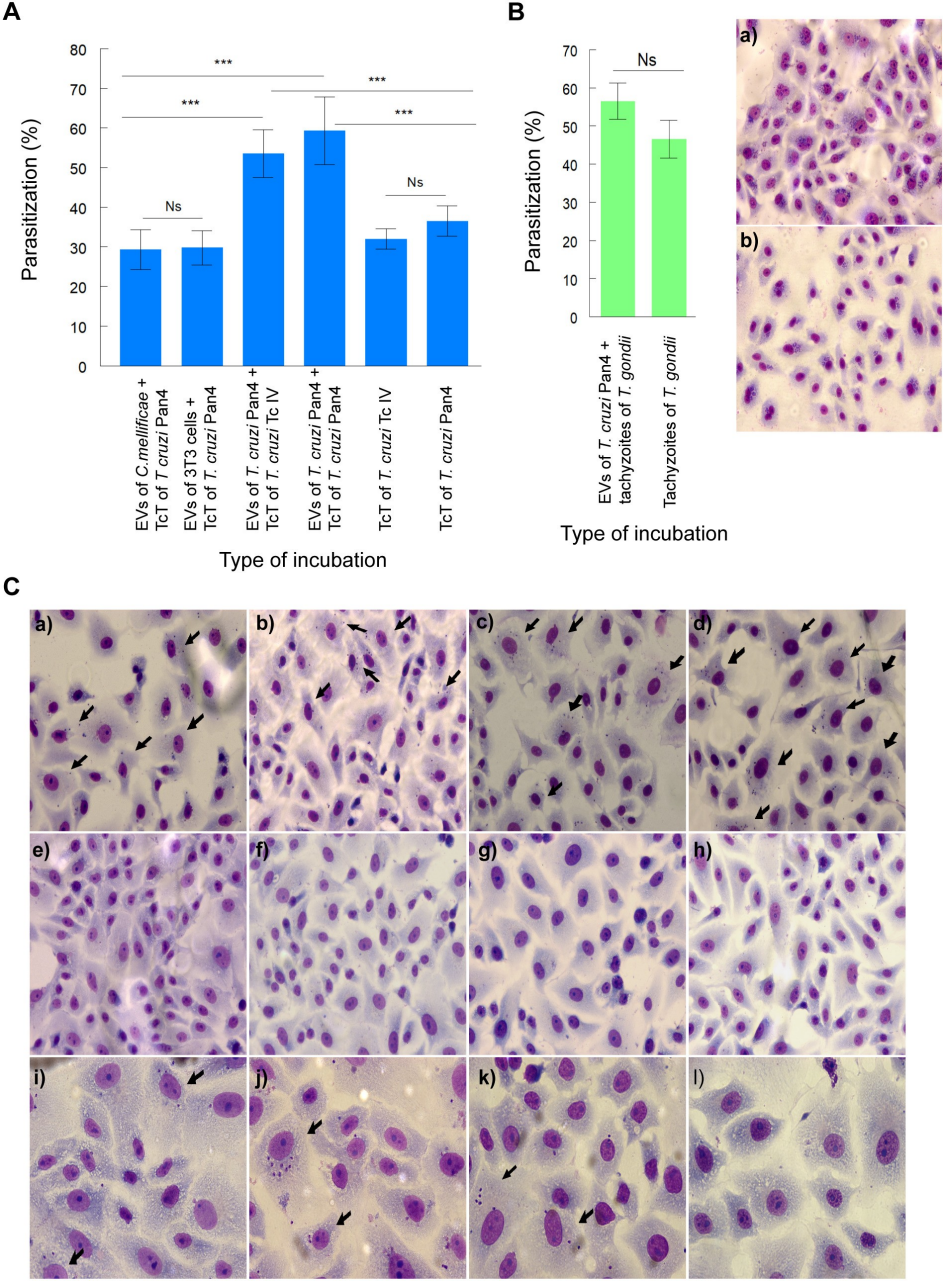
Invasion assays in Vero cells pre-incubated with EVs. The specificity of the effects of EVs of *T*. *cruzi* in increasing the percentages of parasitization is shown in A. The percentages of Vero cell parasitization after the incubation with EVs of *C*. *mellificae* and 3T3 cells and the latter infection with trypomastigotes of *T*. *cruzi* Pan4 were also calculated. In this case, the incubation of cells with EVs from another source different than *T*. *cruzi* did not generate an increase in the percentage of infected cells or in the parasitization indexes, as happens when cells are in contact with EVs of *T*. *cruzi* prior to the infection. In these experiments, the parasitization indexes obtained were 1.31 ± 0.08 in the case of the cells incubated with EVs of *C*. *mellificae*, 1.79 ± 0.28 in the case of the cells incubated with EVs of the 3T3 cell line and 1.54 ± 0.31 in the case of the cells infected with TcT without the incubation with EVs, but it rose to 2.60 ± 0.14 when the cells were previously incubated with EVs of *T*. *cruzi*. Moreover, the percentage of Vero cell parasitization after the incubation with EVs of *T*. *cruzi* Pan4 and the latter infection with trypomastigotes of *T*. *cruzi* 4162 was evaluated. This Fig also shows that these increases in the percentages of parasitization and the parasitization indexes of the cells don´t depend on the strain of *T*. *cruzi* employed to infect the cells, as the percentage of infected cells with the strain 4162 almost doubled the percentage of infected cells not incubated previously with the EVs (parasitization indexes: 2.10 ± 0.14 vs 1.39 ± 0.21, respectively). Vero cells were also incubated for 2 h with EVs of *T*. *cruzi* Pan4 prior to the infection with tachyzoites of *T*. *gondii* RH (B). Results show a slight, non significant increase in the percentage of parasitization in the case of the cells incubated with EVs of *T*. *cruzi* prior to the infection with the tachyzoites, when compared to the cells only infected with the parasite (56.50 ± 4.80 vs. 46.50 ± 4.93); a) Giemsa stain of Vero cells incubated with EVs of *T*. *cruzi* Pan4 and infected with tachyzoites of *T*. *gondii* RH; b) Giemsa stain of Vero cells infected with tachyzoites of *T*. *gondii* RH (low magnification fields, 20x). Images that illustrate the effect of *T*. *cruzi* EVs in increasing the percentage of parasitization of Vero cells by Giemsa stain are shown in C. Vero cells were incubated with EVs of *C*. *mellificae* (a), EVs of 3T3 cells (b) and EVs of TcT of *T*. *cruzi* Pan4 (c, d) and then infected with TcT of *T*. *cruzi* Pan4 (a, b, d) of TcT of *T*. *cruzi* 4162 strain (c). Cells incubated only with EVs of *C*. *mellificae* (e), EVs of 3T3 cells (f), EVs of TcT of *T*. *cruzi* Pan4 (g) and control cells (without incubation) (h) were also included. Low magnification fields (20x) were taken in order to show the overall effect of the different treatments on the cell culture. A magnification field of 40x is included for the incubation of cells with EVs of *C*. *mellificae* and then infected with trypomastigotes of the Pan4 strain (i), cells incubated with EVs of TcT of *T*. *cruzi* Pan4 and then infected with trypomastigotes of the Pan4 strain (j), cells infected only with TcT of *T*. *cruzi* Pan4 (k) and cells only incubated with EVs of TcT of *T*. *cruzi* Pan4 (l). Black arrows illustrate some Vero cells infected with *T*. *cruzi*. The values are the mean percentages ± SEM. Tukey-Kramer test, p<0.0001 (***); Ns: non-significant differences.

Finally, Fig 1A, 1B and 1C show the effect of the incubation of cells with EVs of *C*. *mellificae* and 3T3 cells prior to the infection with *T*. *cruzi* Pan 4 and the increase in the infection of cells when these are incubated with EVs isolated from trypomastigotes of the Pan4 strain and then infected with TcT of the 4162 strain or tachyzoites of *T*. *gondii* RH. In Fig 1A is possible to observe that the incubation of cells with EVs from another source different than *T*. *cruzi* did not boosted an increase in the percentage of infected cells as it happens when the cells are in contact with EVs of *T*. *cruzi* Pan4 prior to the infection. When the cells were incubated with EVs of *C*. *mellificae* and 3T3 cells, the percentage of infected cells obtained were 29.3 ± 5.0 and 29.8 ± 4.3, respectively. These results did not differ significantly from the results obtained in the case of the cells infected only with TcT of *T*. *cruzi* Pan4 without the previous treatment with the EVs (36.5 ± 3.8). In these experiments, the parasitization indexes obtained were 1.31 ± 0.08 in the cells incubated with EVs of *C*. *mellificae*, 1.79 ± 0.28 in the cells incubated with EVs of the 3T3 cell line and 1.54 ± 0.31 in the cells infected with TcT without the prior incubation with EVs, but it rose to 2.60 ± 0.14 when the cells were previously treated with EVs of *T*. *cruzi* Pan4. This Figure also shows that this increase in the parasitization percentage and index of cells is independent of the strain of *T*. *cruzi* employed to infect the cells, as we proved that the infection with TcT of a strain that is classified as Tc IV almost doubled the percentage of infected cells not incubated previously with the EVs (53.5 ± 6.0 vs 32 ± 2.6; parasitization indexes: 2.10 ± 0.14 vs 1.39 ± 0.21, respectively).

In the case of the cells treated with EVs of *T*. *cruzi* Pan4 prior to the infection with tachyzoites of *T*. *gondii* RH, in Fig 1B is possible to observe a slight, non-significant increase in the percentage of infected cells in those incubated with EVs of *T*. *cruzi* prior to the infection with tachyzoites, when compared to the cells infected with the parasite without the previous incubation with the EVs of *T*. *cruzi* (56.50 ± 4.80 vs. 46.50 ± 4.93).

### EVs of *T*. *cruzi* induce cell permeabilization of Vero and HL-1 cells

**Fig 2:**
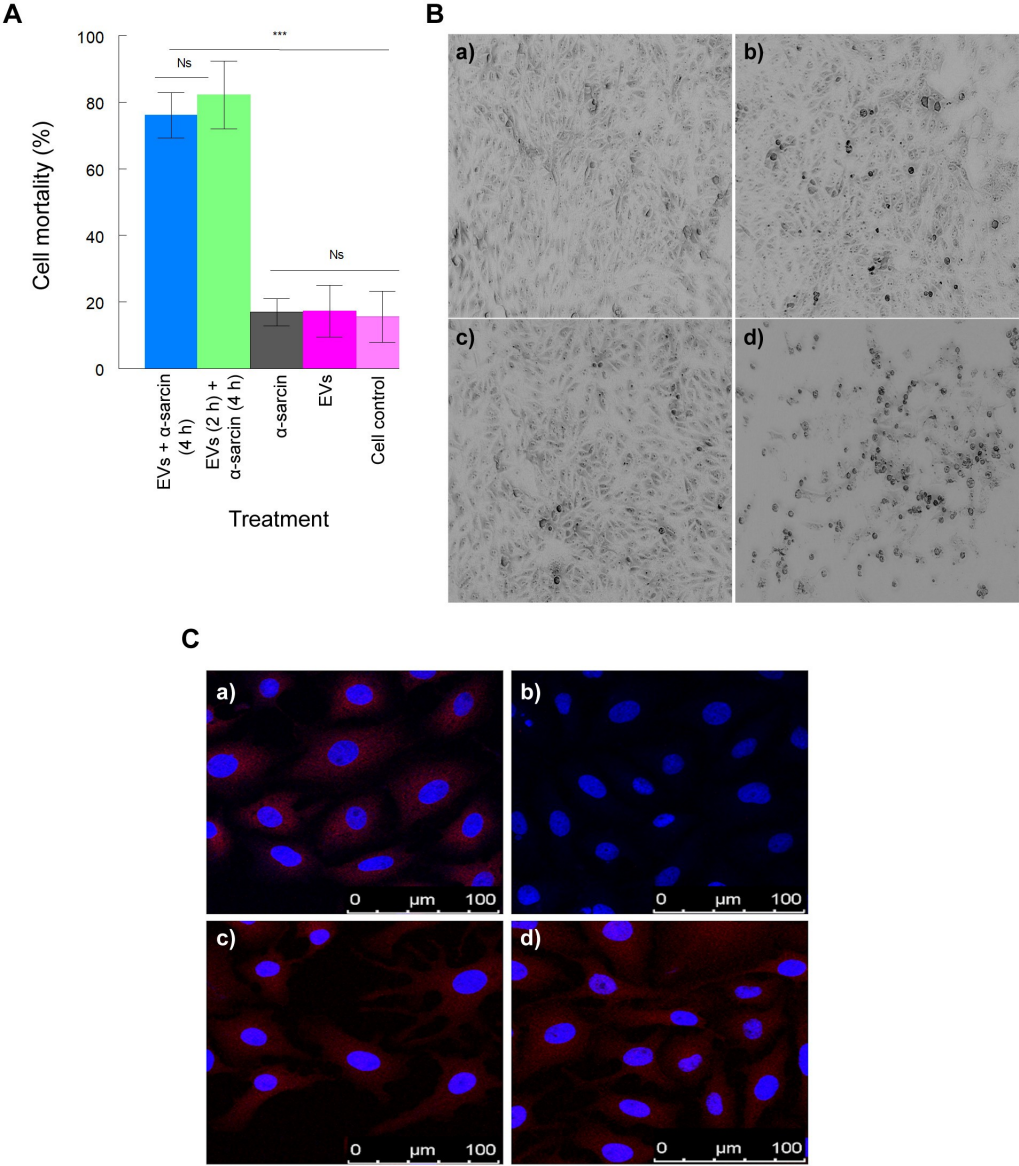
Cell permeabilization of Vero and HL-1 cells by EVs of *T*. *cruzi* Pan4. A cell-viability assay to evaluate the entry of α-sarcin in Vero cells after the incubation with EVs of *T*. *cruzi* using MTT was performed (A). Cells were incubated with *T*. *cruzi* EVs for 2 h, washed, and then incubated with the toxin α-sarcin for 4 h. The MTT cell viability assay was performed at 24 h of the incubation with EVs, following the manufacturer’s instructions. Cells treated only with α-sarcin, EVs and untreated cells were used as controls. The cell monolayers incubated with EVs, α-sarcin or both are shown in B; a) control Vero cells; b) Vero cells treated with EVs for 2 h; c) cells treated with α-sarcin (20 μM) for 4 h; d) cells treated with EVs for 2 h and then treated with α-sarcin (20 μM) for 4 h. The cell cultures were examined at 24 h of the treatment. Low magnification fields were taken in order to show the overall effect of the different treatments on the cell culture. Permeabilization was also evaluated in HL-1 mouse cardiomyocyte cells (C); a) HL-1 mouse cardiomyocyte cells incubated with *T*. *cruzi* EVs for 2 h, fixed in a solution of paraformaldehyde/glutaraldehyde for 1 h and incubated with the anti-β2-adrenergic receptor antibody (antiβ2R); b) HL-1 mouse cardiomyocyte cells not incubated with the EVs, fixed with paraformaldehyde/glutaraldehyde for 1 h and incubated with the anti-β2R (control); c) HL-1 mouse cardiomyocyte cells not incubated with the EVs, fixed with paraformaldehyde/glutaraldehyde for 1 h and permeabilized in a solution of NP-40 in 10 mM citric acid (pH 6) prior to the incubation with the antiβ2R; d) HL-1 mouse cardiomyocyte cells incubated with *T*. *cruzi* EVs for 2 h, fixed with paraformaldehyde/glutaraldehyde for 1 h, permeabilized in a solution of NP-40 in 10 mM citric acid (pH 6) and incubated with the (antiβ2R) (control). The values are the mean percentages ± SEM. Tukey-Kramer test, p<0.0001 (***); Ns: non-significant differences.

A toxin of *Aspergillus giganteus*, α-sarcin, constitutes a ribotoxin with a molecular weight of 16.8 kDa that acts at the ribosomal level, inhibiting the protein synthesis. This toxin has been used to determine the potential permeabilization induced in the cells on the entry of certain viruses [29]. The cells were treated in two ways: i) they were simultaneously incubated with EVs of *T*. *cruzi* Pan4 and α-sarcin or ii) they were incubated with EVs of *T*. *cruzi* Pan4 for 2 h and then with α-sarcin for 4 h. After 24 h of the treatment, the trypan blue exclusion assay was performed. Vero cells treated with EVs and α-sarcin registered mortality percentages of 76.10 ± 6.81 (simultaneous incubation) and 82.20 ± 10.17 (separated incubation of EVs and the toxin). The control cells incubated only with the toxin or with the EVs showed mortality percentages of 16.90 ± 4.10 and 17.23 ± 7.73, respectively, while the percentage of viability of the untreated control culture cells was 15.56 ± 7.67. These results were confirmed using the MTT cell-viability assay, as shown in Fig 2A. In this case, the percentage of viability of the cells treated with EVs and the toxin was 37.36 ± 15.39, while the same percentages in the cells treated only with the toxin or the EVs alone were 90.10 ± 7.03 and 95.30 ± 3.30, respectively. The percentage of viability in the untreated control cell cultures was 99,0 ± 0.1. Fig 2B shows the appearance of the different cell monolayers preincubated with EVs and treated with α-sarcin and the different control cells at 24 h of the treatment.

The beta-2 adrenergic receptor (β2 adrenoreceptor), also known as ADRB2, is a cell membrane-spanning beta-adrenergic receptor that binds epinephrine or adrenaline, whose signalling, via a downstream L-type calcium channel interaction, mediates physiological responses such as smooth-muscle relaxation and bronchodilation [30]. Using an antibody that recognizes an epitope between the amino acids 340–413 (which corresponds to the intracytoplasmic domain of the receptor), we demonstrated that the EVs of *T*. *cruzi* can alter and permeabilize the cell membrane, exposing the epitope to the antibody, as shown in Fig 2C. In the case of the untreated cells, this effect was not detected. An image similar to that of the cells treated with the EVs resulted in the cultures that, prior to the incubation with the antibody, were permeabilized with a solution containing NP-40.

### EVs of *T*. *cruzi* induce the intracellular mobilization of Ca^2+^

**Fig 3:**
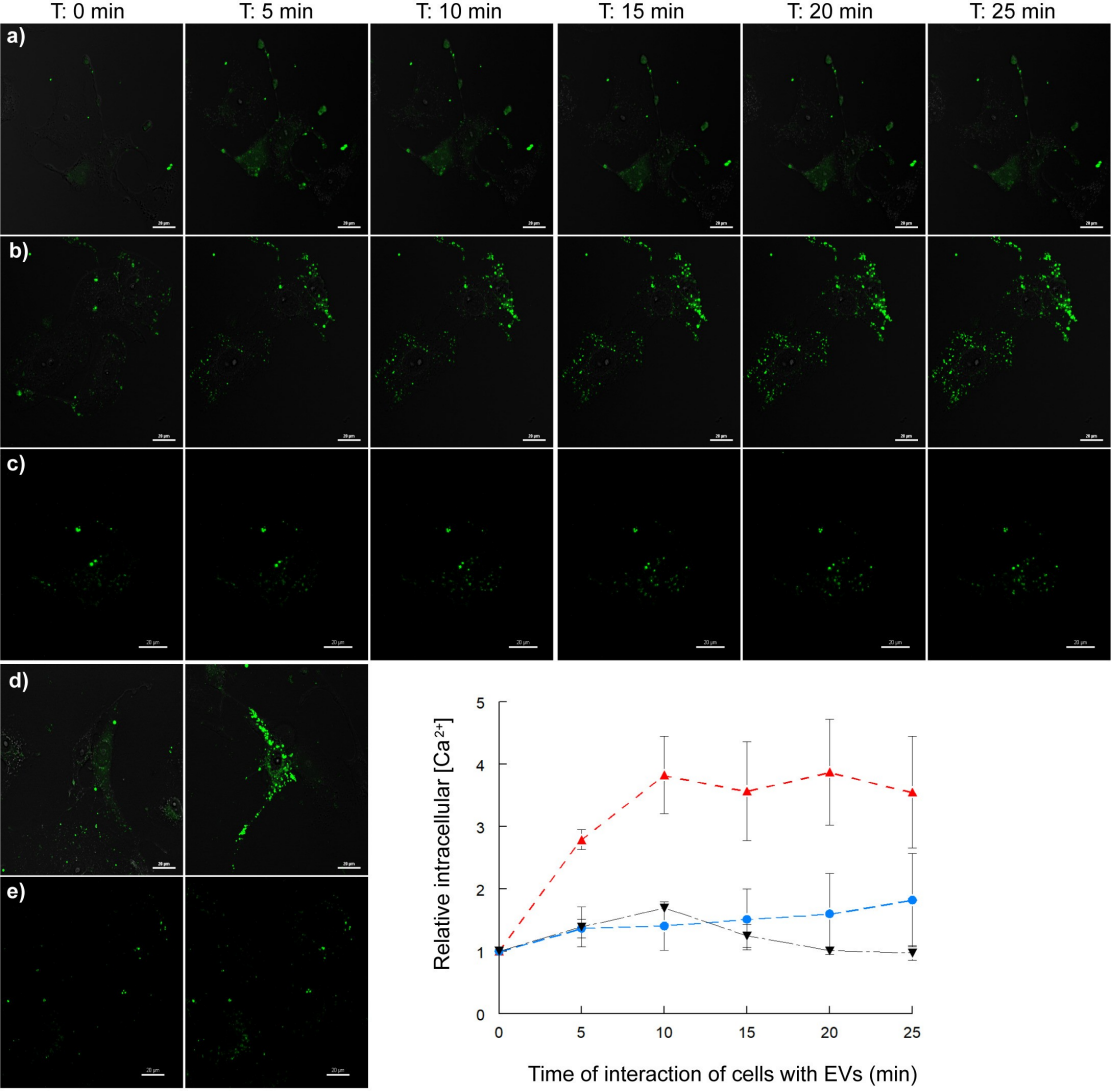
Analyses of the intracellular Ca^2+^ mobilization in Vero cells incubated with EVs of *T*. *cruzi*. Cells were incubated with Fluo4-AM, washed, and then incubated with EVs of *T*. *cruzi*, with examination every 5 min using confocal microscopy. When the cells were incubated in a culture medium with Ca^2+^ and Mg^2+,^ the fluorescence levels progressively increased up to 3.83 ± 0.62 times the initial values as soon as 10 minutes of the interaction (red line). At the same time point, when the cells were incubated with EVs in a culture medium depleted of Ca^2+^, there was an increase of 1.40 ± 0.39 times in the fluorescence levels when compared to the time 0 (blue line). Also, when the cells were incubated with 2.5 μM EDTA, a calcium chelator, there was a 1.69 ± 0.01 increase in the fluorescence levels at 10 min of incubation, when the fluorescence intensity started to decrease and it was possible to observe a more granulated pattern of fluorescence in the cytoplasm (black line). The calcium ionophore A23187 and the cAMP phosphodiesterase inhibitor IBMX were employed as the positive controls of the experiment. A23187 prompted a 64.80-fold rise in the fluorescence levels at 25 min and IBMX prompted a 2.37-fold rise in the fluorescence levels at 10 min of incubation.

The time-course measurements of the intracellular calcium levels of Vero cells treated with the EVs of *T*. *cruzi* Pan4 are shown in Fig 3. Results show that, when the cells were incubated in a culture medium with Ca^2+^ and Mg^2+^, the fluorescence levels increased up to 3.83 ± 0.62 times the initial values as soon as 10 minutes of interaction. Moreover, when the interaction was performed in a culture medium depleted of Ca^2+^ and Mg^2+^, there was also a progressive increase in the fluorescence levels. For example, at 10 min of the interaction, there was a 1.40 ± 0.39-fold increase in the fluorescence levels of the cells, which could correspond to a mobilization of the ions from their intracellular deposits to the cytoplasm.

In the case of the cells incubated in MEM with EDTA, a calcium chelator, there was also a 1.69 ± 0.01 increase in the fluorescence levels at 10 minutes of incubation, when the fluorescence intensity started to decrease. At this point, it is possible to observe a different pattern of distribution of the fluorescence, with the appearance of a more granulated cytoplasm.

The calcium ionophore A23187 was used as the control for the assays of the cells incubated in the medium containing Ca^2+^ and Mg^2+^ and prompted a 64.80-fold rise in the fluorescence levels at 25 min. The cAMP phosphodiesterase inhibitor, 3-isobutyl-1-methylxanthine (IBMX) was used as the control for the induction of ion output from the intracellular Ca^2+^ deposits to the cytoplasm and prompted a 2.37-fold rise in the fluorescence levels at 10 min.

### EVs of *T*. *cruzi* disrupt the actin cytoskeleton

**Fig 4:**
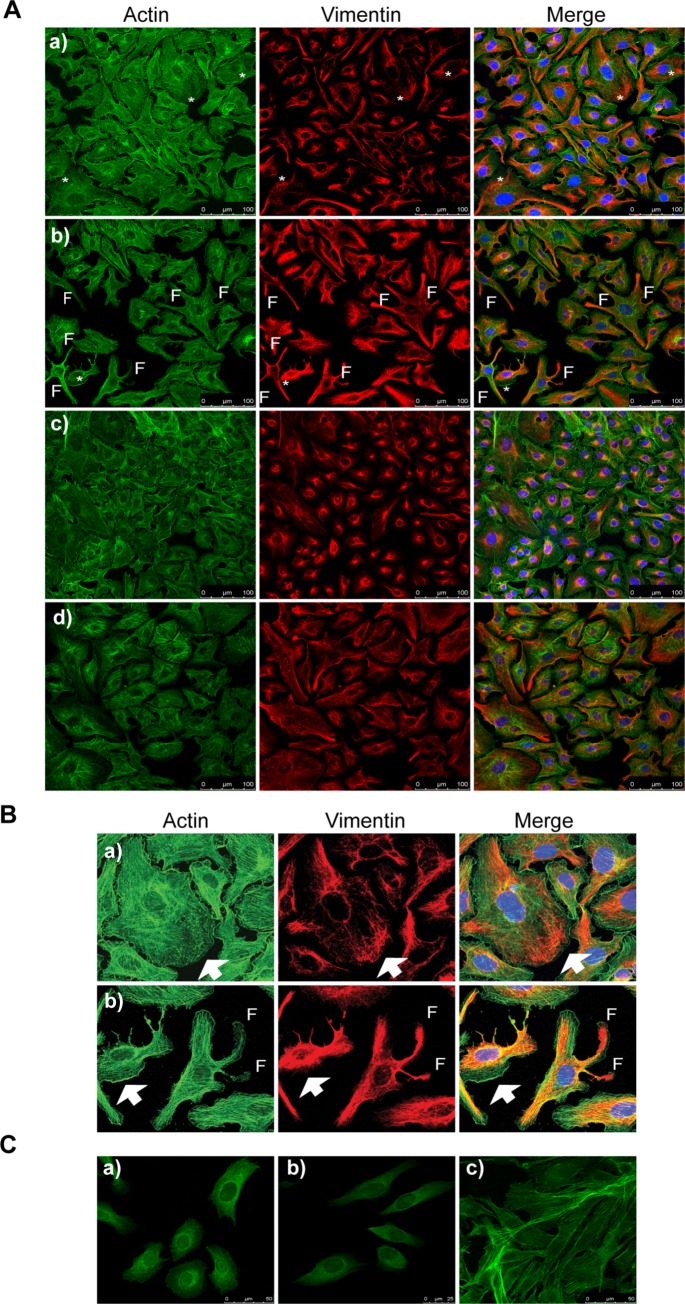
Disruption of the actin cytoskeleton and formation of filopodia in Vero cells incubated with EVs of *T*. *cruzi*. Vero cells were incubated with EVs of *T*. *cruzi* Pan4 during different time points (A). The disruption of the actin filaments is observed since 15 minutes of treatment of the cells with EVs, the formation of filopofia is more evident at 30 min of treatment (*); a) Vero cells incubated with EVs of *T*. *cruzi* Pan4 for 15 min; b) Vero cells incubated with EVs of *T*. *cruzi* Pan4 for 30 min. Vero cells incubated with the supernatant of the EVs purification medium (c) and Vero cells without the EVs treatment (d) were employed as controls of the experiment. A magnification is shown in B; a) Vero cells incubated with EVs of *T*. *cruzi* Pan4 for 15 min; b) Vero cells incubated with EVs of *T*. *cruzi* Pan4 for 30 min. In this magnification, white arrows indicate the disorganization of actin filaments and F the formation of filopodia in the cells. The disruption of the actin cytoskeleton in Vero cells incubated with EVs of *T. cruzi* for 120 min is shown in C; a) and b) Vero cells incubated with EVs of *T*. *cruzi* Pan4 for 120 min and c) Vero cells without the incubation with EVs.

The analysis of the effect of the EVs of *T*. *cruzi* Pan4 over the actin cytoskeleton is shown in Fig 4A, 4B and 4C. The disorganization of the actin filaments was visible in the cells showing greater globular actin (GA) from 15 min up to 120 min after the treatment with EVs (Fig 4A, 4B and 4C). On the other hand, vimentin appeared to withdraw from the areas in the cytoplasm where the actin is disorganized and concentrated in the parts of the cytoplasm where GA is less patent. The morphology of the treated cells appeared to be altered and filopodia (F) were visible, giving a dendritic aspect to the cell. These effects were reversible 24 h after the treatment; both the cytoskeleton images and the morphology of the cells incubated with EVs were similar to that of the control cells not treated with EVs.

### EVs of *T*. *cruzi* arrest of the cell cycle in phase G0/G1

**Fig 5:**
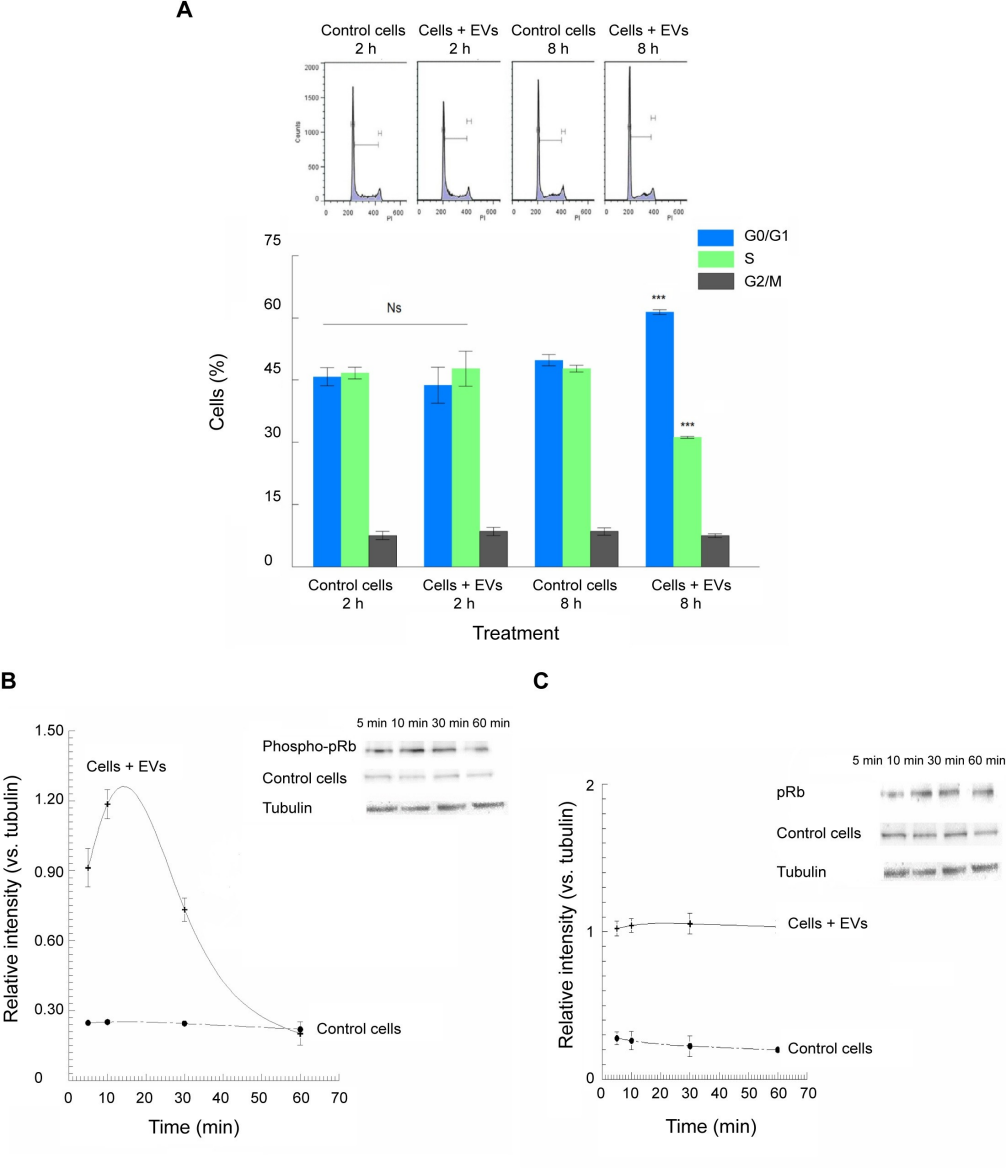
Arrest of the cell cycle by EVs of *T*. *cruzi* Pan4. Vero cells were incubated with EVs of *T*. *cruzi* Pan4 for 1 h, and then washed and incubated for 2 and 8 h, when the flow cytometric analyses were performed (A). At 8 h it is possible to observe an increase in the percentage of cells at phases G0/G1 and a decrease in the percentage of cells at phase S. The phosphorylation of the pRb in Vero cells incubated with EVs of *T*. *cruzi* Pan4 (B) and the expression of the non-phosphorilated pRb in Vero cells incubated with EVs of *T*. *cruzi* Pan4 were also evaluated (C). The values are the mean percentages ± SEM. Tukey-Kramer test, p<0.0001 (***); Ns: non-significant differences.

The influence of the EVs of *T*. *cruzi* Pan4 on Vero cells cycle was analysed with Vero cells previously synchronized in the S phase and treated as described in Methods. The changes in the cell cycle were analysed by flow cytometry 2 and 8 h after the addition of the EVs. Fig 5A shows the percentage of cells in the different phases of the cell cycle. At 8 h after the addition of the EVs, the percentage of cells increased at phases G0/G1 and decreased at phase S.

Fig 5B and 5C show the results of the levels of the protein of retinoblastoma (pRb) in its phosphorylated and non-phosphorilated states, in the cells treated with EVs of *T*. *cruzi* Pan4 and the untreated control cells. The protein expression increased for the phosphorylated pRb from the first minutes of the interaction of the EVs with the cells, reaching the maximum phosphorylated state at 15 min of the interaction and declining to values similar to those of the control cells at 60 min of treatment.

## Discussion

The cell-invasion process of *T*. *cruzi* has been widely studied. Numerous mechanisms are known to be involved in preparing the cell to induce the endocytosis of trypomastigotes into non-phagocytic cells [31–36] and among the natural agents that induce massive entry and cellular infection are the EVs secreted by trypomastigotes.

EVs from trypomastigotes of the Pan4 strain were first isolated in 2016, when de Pablos et al. confirmed that the C-terminal region of MASPs proteins is present in the EVs secreted by the trypomastigotes derived from tissue-culture cells [18]. Following the methodology described above using differential centrifugation and then Nanoparticle Tracking analysis, we obtained an homogeneous population of EVs under our experimental conditions. Also, the yield was higher than that of other authors using others strains or forms of the parasite. Trocoli Torrecilhas et al. (2009) employed 5 μg of protein from 1x10^5^ trypomastigotes of the Y strain [15]. Garcia Silva et al. (2014) have reported a protein yield of the vesicular fraction of 1.2 μg per 1x10^10^ epimastigotes from the DM28c strain [14]. In our case, we found 12 μg of protein of EVs after the incubation of 1x10^7^ trypomastigotes of the Pan4 strain for 5 h at 37°C in the culture medium for the release of EVs.

To track the time course of the effect of EVs in increasing cell parasitism, we performed infections at different times after incubating the cells with EVs and we found that the cells are still susceptible to increased parasitism at least for 8 h after the treatment (p <0.0001). However, 24 h after the incubation with the EVs, the percentage of infected cells in the treated and non-treated cultures were similar (S2B Fig). On the other hand, we observed that increasing amounts of EVs can increase the percentage of infected cells with sigmoidal kinetics, reaching a maximum of 89% infected cells (0.5 μg/mL) and ED50 of 0.38 μg/mL, a dose that was employed in subsequent experiments. In 2014, Garcia Silva et al. observed that the treatment with small amounts of EVs (160 ng) can prevent cells from appearing oversaturated in a tRNA^Glu^-derived 5′ halves visualization analysis by FISH [14]. In the same study, the authors determined that 30 min after the treatment of cells with EVs, a diffuse fluorescent cytoplasmic pattern appeared, which becomes granular after 2 h of treatment. Regarding the interaction and posterior infection of the cells after the treatment with the EVs, Cestari et al. (2012) pre-incubated Vero cells for 30 min before adding the trypomastigotes of the Sylvio X10/6, DTU I strain [17]. This reflects that the incubation conditions with respect to the amount of EVs used and the incubation time vary among the different research groups, and it should be taken into account that the conditions selected by a researcher do not necessarily correspond to the conditions of a natural infection [14].

It has been demonstrated *in vitro* (in non-phagocytic cells and monocytes), as well as *in vivo*, that EVs of *T*. *cruzi* increase the number of infected cells [15,17,37–38]. Under our experimental conditions, cells pretreated with EVs of *T*. *cruzi* Pan4 registered infection percentages of over 3.5-fold higher than for cells infected without the prior incubation with the EVs. The parasitization index (number of parasite per cell) was also two-fold that of the parasitization index of the control infected cells without prior treatment. In 2009, Trocolli Torrecilhas et al. reported that *T*. *cruzi* trypomastigotes invade 5-fold more susceptible cells when these were preincubated with purified parasite EVs [15]. Cestari et al. (2012) also demonstrated that THP-1 derived plasma membrane vesicles (ectosomes) could simultaneously induce an increase in Vero cell invasion [17]. This invasion was dose dependent, non-specific for parasite strain or eukaryotic cell line, and dependent on the parasite infective stage. We also proved that the increase in parasitization is specific to *T*. *cruzi* trypomastigotes but non-specific for the parasite strain and that the incubation of cells with EVs from another trypanosomatid species or those from eukaryotic cells didn´t increased the percentages of parasitization.

The interaction EVs-cell and the latter activity of EVs appears to depend on their binding through lectins to the plasma membrane and on a presumably enzymatic protein activity, given that the thermal and chemical treatments of EVs with trypsin, proteinase K, and sodium periodate drastically reduced parasitism of the cells with which they interacted. During the adhesion and invasion process of T to the host cells, a number of glycosylated molecules are expressed on the surface of the parasite. Examples include mucins, trans-sialidases, MASPs and the gp85 family of proteins [35]. Glycosylated proteins have also been detected in EVs by proteomic analyses [9,39] some with important activities and biological significance, such as trans-sialidases (TS/SAPA, TC85, gp82, gp90, CRP) [40], cruzipain [41], gp63, MASPs [42], and other types of mucins [43]. As these proteins are located on the surface of the EVs, they can bind specifically to the proteins in the plasma membrane and this would explain why the reduction of the carbohydrates of the EVs after the sodium periodate treatment can affect the binding of EVs to the surface of the cells. On the other hand, some of these glycoproteins have enzymatic activities essential for the interaction of trypomastigotes during the invasion process and maybe in the interaction of EVs with the plasma membrane.

It has been reported that the infection with some type of viruses lead to a permeabilization mechanism where the plasma membrane allows the entry of some high molecular weight molecules such as α-sarcin (16.8 KDa) [44–46], toxin that lacks a membrane receptor, unlike other toxins that affect the protein synthesis and are internalized via endocytosis [45,47]. The same effect was observed incubating cells with EVs of trypomastigotes of *T*. *cruzi*, as our results demonstrate that they can permeabilize Vero and HL-1 cell lines. Cell counts with trypan blue 24 h after the preincubation of cells with EVs and subsequent incubation with α-sarcin registered as much as 76.10% mortality. The control cells incubated only with α-sarcin, only with EVs, and without either showed percentages of cell death of 16.90%, 17.23%, and 15.56%, respectively. The percentages of mortality (100 - % of viability in Fig 2) of the cells using MTT were 62.64% in the case of the cells previously incubated with EVs and 9.90% in cells incubated only with the toxin. In 1990, Castanys et al. have reported that infective metacyclic forms of *T*. *cruzi* secrete a glycoprotein involved in cell permeabilization that enabled the entrance of molecules such as α-sarcin [24].

Permeabilization was also evaluated in HL-1 cardiac muscle cells with confocal microscopy, using an antibody directed to an epitope of the β2-adrenergic receptor located in the intracytoplasmic region of the receptor anchoring. In this experiment, fluorescence was detected in the cytoplasm of cells previously incubated with EVs and in cells not treated with EVs but permeabilized with the detergent NP-40. This permeabilization may be the result of changes in the cell membrane that allow the direct entry of the antibodies into the cytoplasm or by a transient disorganization of the membrane, capable of exposing the antigens present in inside the cell, with consequent exposure to the immune system. The presence of autoantibodies against β-adrenergic receptors in the serum of chagasic patients has been reported [48–51], although authors have related such emergence to the recognition of exposed parts in the membrane due to cross reactivity to ribosomal acidic proteins P0 of the parasite [52]. A study related to the recognition of epitopes of the β-adrenergic receptors inserted into the inner side of the membrane by autoantibodies would be necessary to confirm the possible hypothesis that the permeabilization of the cardiac cells by the parasitic EVs lead to the exposure of these receptors to the immune system and then elicit the production of autoantibodies.

The recognition and the invasion processes of the trypomastigotes to the host cells involve molecules over the surface of both the trypomastigote and the host cell. A ligand-receptor recognition occurs and generates in both a series of events that raises the intracellular Ca^2+^ levels due to the mobilization of the ions from the endoplasmic reticulum and the mitochondria [53–57]. This boost in calcium levels is also responsible for higher cAMP levels [58], facilitating the release of EVs and their later fusion with the plasma membrane [59]. The treatment of Vero cells with EVs of *T*. *cruzi* Pan4 raised cytoplasmic Ca^2+^ levels and to determine the kinetics and origin of these Ca^2+^ ions, we treated cells with EVs and studied the result every 5 min under confocal microscopy. From 5 min of the treatment, fluorescence intensified in both culture media (with and without calcium). This implies that the contact of Vero cells with EVs raises cytoplasmic Ca^2+^ levels that could come both from the intracellular deposits of calcium and the extracellular medium. The fluorescence pattern detected resembles the one in control cells treated with the xanthine IBMX for the Ca^2+^ mobilization from the intracellular deposits [60] or when the cells were incubated with the ionophore A23187 [61], a compound that allows Ca^2+^ to enter cells from the culture medium.

It has been demonstrated in different types of eukaryotic cells that the intracellular levels of calcium induce an asymmetric distribution of phospholipids in the plasma membrane by the activation of the enzymes scramblase and floppase. Then, phosphatidylserine and phosphatidylethanolamine are exposed in the outer side of the membrane and contribute to the activation of Ca^2+^-dependent proteases, followed by the release of EVs [62]. The exposure of anionic phospholipids to EVs strengthens the fusogenic properties of these vesicles, which could be a prerequisite for the release of the EV content. This could mean that the higher intracellular calcium levels and the changes in the distribution of phospholipids could explain the permeabilization induced in the host cell after the treatment with the EVs of *T*. *cruzi*, allowing the entrance of a toxin of ~17 kDa such as α-sarcin. Moreover, increases in intracellular Ca^2+^ could trigger a greater release of EVs from the cells exposed to the parasite [3,17,63], as more calcium prompts a strong response of EV release in other cell lines [64–65].

Calcium ions also contribute to the reorganization of the cytoskeleton through the activation of cytoplasmic proteins such as calpain and gelsolin. These proteins cut the actin cytoskeleton protein network, allowing membrane budding and removing capping proteins at the end of the actin filaments [66–67]. A disruption in actin filaments and vimentin at the time of the invasion of cells with trypomastigotes has been demonstrated [68–69] using drugs like cytochalasin B and latrunculin, which affect the cytoskeletal structure and functions and, therefore, the entrance of the parasite in non-phagocytic cells [32–33,70]. It has been mentioned that the increase in intracellular Ca^2+^ leads to a rapid and transient reorganization of host-cell microfilaments, including the disassembly of the actin cytoskeleton, which is important for the entry of T into the host cells [71–73]. Studying the gene-expression changes caused by microvesicles of *T*. *cruzi* epimastigotes of the DM28c strain in mammalian host cells, Garcia Silva et al. (2014) observed an induction of a broad response, including the modification of the host-cell cytoskeleton and the extracellular matrix [74]. In fact, the regulation of actin cytoskeleton is one of the pathways identified as being affected by EV treatment in the profile of transcriptome changes [74]. Noting increased fluorescence in cells incubated with EVs in the presence of Fluo-4AM, we suspected that these changes in the Ca^2+^ mobilization induced by EVs could directly affect the actin cytoskeleton, as happens when the parasite begins to invade the host cell. Our results showed a clear disruption of host-cell actin from 15 min after the incubation with EVs, an effect that remains at 120 min but not 24 h after the treatment with EVs. Ferreira et al. (2006) indicated that different strains of Mt of *T*. *cruzi* can invade host cells through both actin cytoskeleton-dependent and independent routes, by engaging different surface molecules for attachment while triggering different signal-transduction pathways [34]. For example, host-cell invasion by the strain CL Mt, mediated mainly by the surface molecule gp82, is associated with F-actin disassembly whereas the G strain is gp35/50-mediated invasion by strain G depends on target-cell actin cytoskeleton [34,75].

The analysis of the cell cycle events revealed how at 8 h after the addition of the EVs the percentage of cells in each of the cycle phases significantly differed when compared to control values, showing an arrest of the cell cycle in the G0/G1 phases. Previous observations regarding the *in vitro* life cycle of *T*. *cruzi* in cultured cells demonstrated a low cell-division rate among cells infected with the parasite [76–78]. In this regard, Ca^2+^ may be responsible for the cell cycle changes, as they act as second messengers in the control of the cell cycle. Thus, Ca^2+^/calmodulin activate the complex CDK4/cyclin D1, which regulates the protein of Retinoblastoma (pRb1), the main inhibitor of the DNA synthesis [79]. From our results, it is evident that the phosphorylation of the protein Rb takes place from the first few min of the EVs/cell interaction. Here, phosphorylated pRb increased rapidly, while in the cells without the treatment with EVs no such change was detected (Fig 5B and 5C). However, at 60 min of treatment with EVs, these increases in phosphorylation returned to normal levels. This apparently arrested synthesis, preventing the cells from entering phase S of the cycle. Moreover, a series of “calcium sensors” present in the cell cytoplasm, such as the stromal interaction molecule 1 (STIM1), is involved in the progression of mitosis. Cells lacking this protein may arrest the cells in phases G0/G1, as occurred in our experiments. This implies that this protein is required for the progression of the cells in the phase of DNA synthesis or phase S [80]. The arrest of cells in the G0/G1 phases exerted by EVs of *T*. *cruzi* Pan4 was possibly caused by increased expression of cyclin-dependent kinase inhibitor p21 and the subsequent decrease of phosphorylated protein (pRb) [81]. The higher intracellular calcium levels were also involved in cell-cycle events. In fact, the indirect role of high levels of calcium in cell arrest in these phases has been examined by Wu et al. (2006) [82], who employed capsaicin and blocked the cell cycle in the previous phase of DNA synthesis. This effect was reversed with BAPTA, an intracellular Ca^2+^ chelator. Together with the rises of intracellular calcium levels, and because of these high levels, these researchers have recently questioned the role of actin networks nucleated by the complex Arp2/3 in the signalling events necessary for the progression of the cell cycle in non-transformed cells [82–83] and demonstrated that Arp2/3 is not able to act as a sensor for the start of the phase S in the cell cycle per se, such as the actin filaments. Previous studies have shown that the use of cytochalasin B at very low doses detains the cell cycle in phases G0/G1 as in our experiments with EVs while the inhibitors that act in the polymerization of actin stopped the cell cycle before the cytokinesis [84–86].

In conclusion, it has been shown that the incubation of cells with EVs of TcT of *T*. *cruzi* Pan4 strain induce a number of changes in the host cells that include 1) a change in cell permeability, and 2) higher intracellular levels of Ca^2+^ that can alter the dynamics of the actin cytoskeleton and arrest the cell cycle at G0/G1 prior to the DNA synthesis necessary to complete mitosis. In the end, these changes induced by the EVs aid their invasion of host cells, augment the percentage of cell parasitization, and possibly cause some characteristic manifestations of Chagas disease.

## Supporting information

S1 Fig Evaluation of the isolation procedure of EVs of *T. cruzi*. TEM images show the variety of sizes of EVs of TcT of *T*. *cruzi* Pan4 strain (A). The presence of cruzipain, trans-sialidase and MASPs (SP) in these EVs was also evaluated by Western blotting (B); a) detection of cruzipain in EVs of tripomastigotes of *T*. *cruzi* Pan4 (1) and in a lysate of trypomastigotes of *T*. *cruzi* Pan4 (2); b) detection of trans-sialidase (mAb 39) in a lysate of trypomastigotes of *T*. *cruzi* Pan4 (1) and in EVs of tripomastigotes of *T*. *cruzi* Pan4 (2); c) detection of MASPs (SP) in EVs of tripomastigotes of *T*. *cruzi* Pan4. NTA (C) and DLS (D) of EVs of *T*. *cruzi* Pan 4 strain, DLS of EVs of *Crithidia mellifica*e (E) and DLS of EVs of the 3T3 cell line (F) are also included.
(TIF)

S2 Fig Optimization of EV-cell incubation conditions and invasion assays. Vero cell parasitization after the incubation with different doses of EVs of *T*. *cruzi* Pan4 was evaluated and the maximum increase in this percentage was achieved when 0.50 μg/mL of EVs were employed (A). The ED50 was calculated and employed in the incubation of the cells with EVs. These cells were subsequently infected with trypomastigotes at different time points and the parasitization percentages were calculated (B). Pictures a) and b) from this Figure show Vero cells incubated with EVs prior to the infection with TcT of the Pan4 strain and stained with Giemsa. Picture c) corresponds to the control cells infected with TcT without the previous treatment of cells with EVs. Additionally, the percentages of parasitization of Vero cells incubated with *T*. *cruzi* EVs submitted to thermal (C) and chemical treatments (D) were also calculated. The thermal treatment appeared to “inactivate” the EVs, as no increase in the percentage of parasitization was detected. In the case of the cells incubated with the chemically-treated EVs, the percentage of parasitization was also lower compared to the percentage of the cells incubated with EVs without treatment. Tukey test, p<0.0001 (***); Ns: non-significant differences.
(TIF)
