# Supplementary figures and images for: Extracellular vesicles of Trypanosoma cruzi tissue-culture cell-derived trypomastigotes: Induction of physiological changes in non-parasitized culture cells
Source: PLoS Negl Trop Dis. 2019 Feb 21;13(2):e0007163. doi: 10.1371/journal.pntd.0007163 (PMC6383987; doi:10.1371/journal.pntd.0007163)

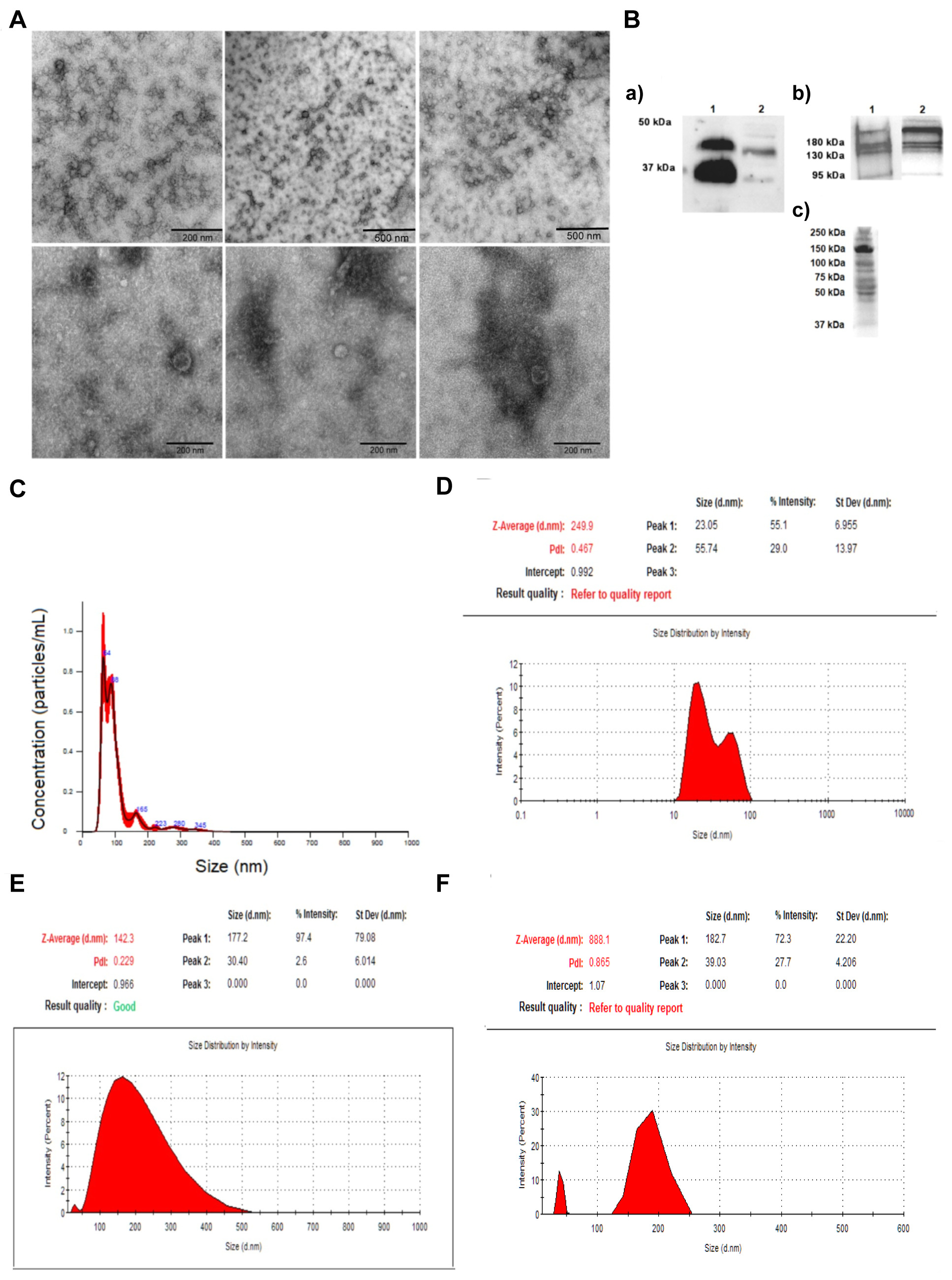

Supplement: S1 Fig — TEM images show the variety of sizes of EVs of TcT of T. cruzi Pan4 strain (A). The presence of cruzipain, trans-sialidase and MASPs (SP) in these EVs was also evaluated by Western blotting (B); a) detection of cruzipain in EVs of tripomastigotes of T. cruzi Pan4 (1) and in a lysate of trypomastigotes of T. cruzi Pan4 (2); b) detection of trans-sialidase (mAb 39) in a lysate of trypomastigotes of T. cruzi Pan4 (1) and in EVs of tripomastigotes of T. cruzi Pan4 (2); c) detection of MASPs (SP) in EVs of tripomastigotes of T. cruzi Pan4. NTA (C) and DLS (D) of EVs of T. cruzi Pan 4 strain, DLS of EVs of Crithidia mellificae (E) and DLS of EVs of the 3T3 cell line (F) are also included. (TIF) [file pntd.0007163.s001.tif]

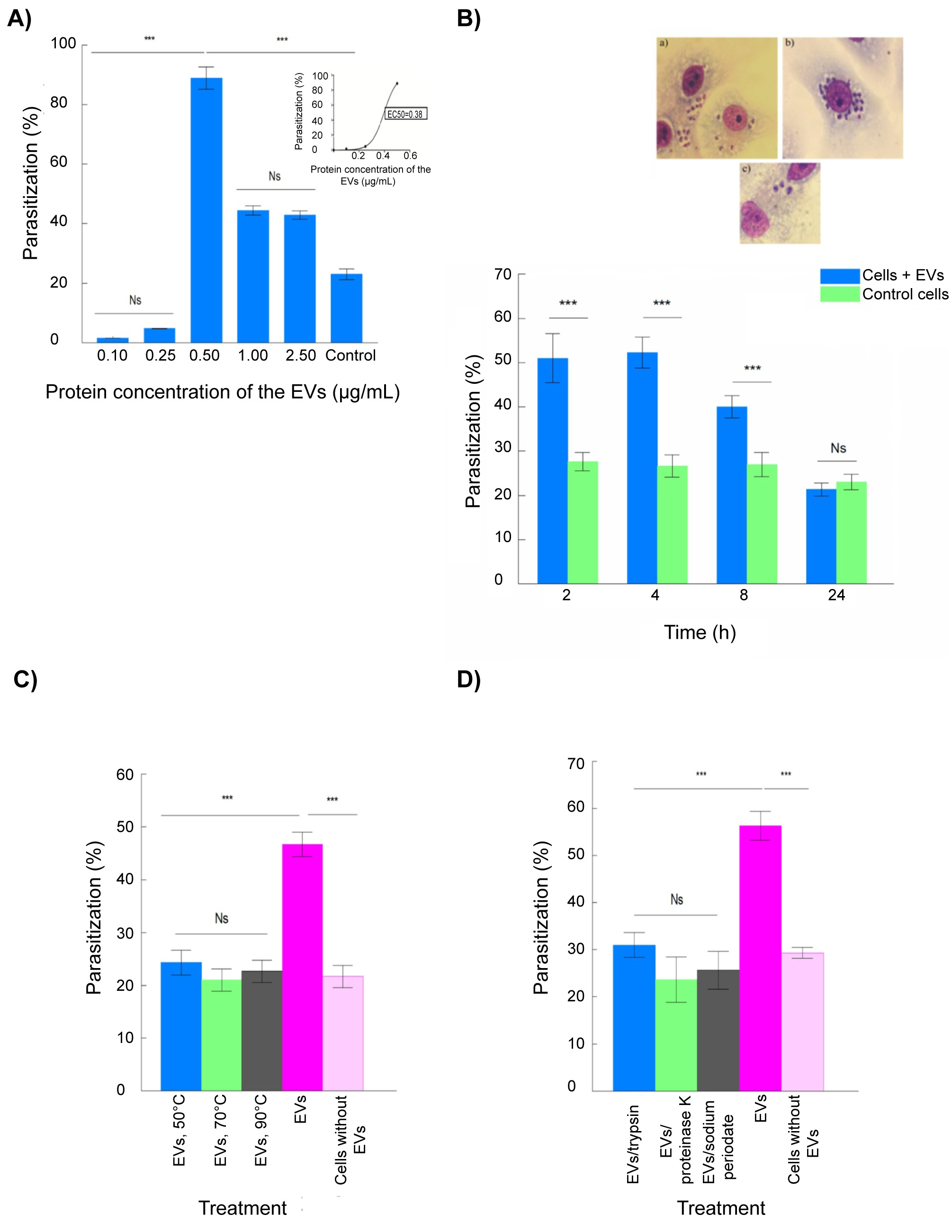

Supplement: S2 Fig — Vero cell parasitization after the incubation with different doses of EVs of T. cruzi Pan4 was evaluated and the maximum increase in this percentage was achieved when 0.50 μg/mL of EVs were employed (A). The ED50 was calculated and employed in the incubation of the cells with EVs. These cells were subsequently infected with trypomastigotes at different time points and the parasitization percentages were calculated (B). Pictures a) and b) from this Figure show Vero cells incubated with EVs prior to the infection with TcT of the Pan4 strain and stained with Giemsa. Picture c) corresponds to the control cells infected with TcT without the previous treatment of cells with EVs. Additionally, the percentages of parasitization of Vero cells incubated with T. cruzi EVs submitted to thermal (C) and chemical treatments (D) were also calculated. The thermal treatment appeared to “inactivate” the EVs, as no increase in the percentage of parasitization was detected. In the case of the cells incubated with the chemically-treated EVs, the percentage of parasitization was also lower compared to the percentage of the cells incubated with EVs without treatment. Tukey test, p<0.0001 (***); Ns: non-significant differences. (TIF) [file pntd.0007163.s002.tif]
